# Stromal marker fibroblast activation protein drives outcome in T1 non-muscle invasive bladder cancer

**DOI:** 10.1371/journal.pone.0257195

**Published:** 2021-09-15

**Authors:** Tim Muilwijk, Murat Akand, Sofie Daelemans, Koen Marien, Yannick Waumans, Mark Kockx, Loïc Baekelandt, Thomas Van den Broeck, Frank Van der Aa, Thomas Gevaert, Steven Joniau

**Affiliations:** 1 Department of Urology, University Hospitals Leuven, Leuven, Belgium; 2 Organ Systems, KU Leuven, Leuven, Belgium; 3 Pathology – Histology, Imaging and Quantification, CellCarta, Antwerp, Belgium; 4 Medical Biochemistry, Faculty of Pharmaceutical, Biomedical and Veterinary Sciences, University of Antwerp, Antwerp, Belgium; 5 Department of Pathology, AZ Klina, Brasschaat, Belgium; Centro Nacional de Investigaciones Oncologicas, SPAIN

## Abstract

Fibroblast activation protein-α (FAP) is a transmembrane peptidase and a surrogate marker for cancer-associated fibroblasts (CAFs). FAP has been linked to worse prognosis and therapy resistance in several cancers. We hypothesised that FAP might have a prognostic 3biomarker potential to stratify patients with high-grade (HG) T1 non-muscle-invasive bladder cancer (NMIBC). We selected 30 patients with HG T1 NMIBC that progressed to ≥T2 disease which were pair-matched based on CUETO progression score variables with 90 patients that did not progress. After revision a final cohort of 86 patients was retained. Slides were stained for FAP, the luminal marker GATA3 and the basal marker CK5. All HG T1 tumour regions of interest (ROIs) within each patient were annotated, analysed and scored using image analysis software. FAP expression in HG T1 ROIs was significantly higher in progressors vs. non-progressors and was prognostic for recurrence-free survival, progression-free survival, cancer-specific survival, and overall survival. FAP expression in HG T1 ROIs remained strongly prognostic for these outcomes in a bivariable model corrected for adequate BCG per FDA definition. Expression of GATA3 and CK5 did not differ between progressors vs. non-progressors, and were not prognostic for these outcomes. FAP might serve as an easily applicable prognostic biomarker to risk-stratify patients with HG T1 NMIBC if these results are prospectively validated in a larger series.

## Introduction

Due to a significant heterogeneity in clinical behaviour of stage T1 non-muscle-invasive bladder cancer (NMIBC), there is an unmet need for prognostic biomarkers to aid in patient counseling and treatment decisions [1]. Fibroblast activation protein-*α* (FAP) is a transmembrane peptidase expressed on stromal cells and acts as a surrogate marker for cancer-associated fibroblasts (CAFs) which play a major role in the tumour microenvironment [2, 3]. FAP overexpression in CAFs has been linked to resistance to therapy in several solid cancers, such as colorectal, pancreatic, gastric and ovarian cancer, highlighting the importance of stromal components in determining outcome [2–5]. Furthermore, FAP overexpression in colorectal, pancreatic and ovarian cancer is associated with an increased risk of lymph node metastasis and worse prognosis [2, 6]. We hypothesised that FAP expression might have a prognostic biomarker potential in patients with T1 NMIBC. To that end, we designed a retrospective study with high grade (HG) T1 NMIBC and pair-matched patients, who did experience progression to ≥T2 disease, with patients who did not progress. Given the emerging data on the correlation between molecular subtypes and clinical outcome in bladder cancer (BC), we also included CK5 and GATA3 as surrogate immunohistochemical (IHC) markers for basal and luminal differentiation, respectively [7, 8].

## Materials and methods

**Fig 1:**
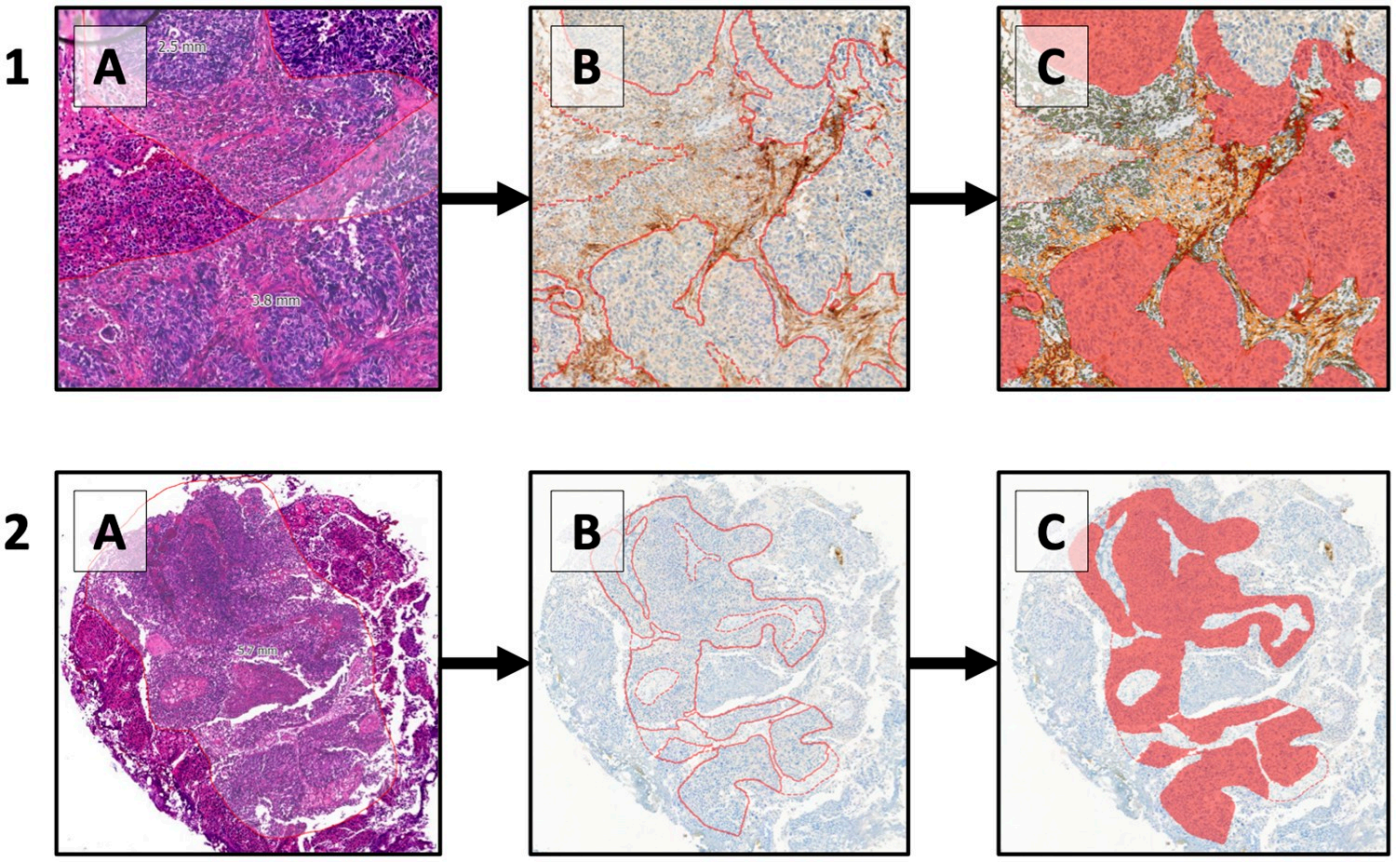
Patient 1 and patient 2: Illustrative figure of the analytical workflow. Patient 1 shows high FAP expression and patient 2 shows absent FAP expression. A: T1 tumour regions of interest (ROI) within each patient were digitally annotated on a serial H&E-stained slide by an expert uro-pathologist (TG) within Pathomation digital pathology software. B: Digital annotations of ROIs were transferred from the H&E slides to the FAP-IHC slides with the image analysis software Visiopharm, and areas with tumour and stroma were separated. C: FAP expression was measured in the stromal areas only as relative marker area (RMA) with integration of low, medium and high expression levels (formula, calculated using Visiopharm: [RMA low FAP] + [RMA medium FAP] + [RMA high FAP]) (red coloured areas are omitted tumour areas).

From our institutional database of HG T1 NMIBC, we retrospectively selected 30 patients who progressed to ≥T2. Those were pair-matched using the Club Urologico Español de Tratamiento Oncologico (CUETO) progression score variables with 90 patients who never progressed despite having even longer follow-up [9]. All patients completed at least 5/6 doses of their initial BCG induction course. Haematoxylin slides and the corresponding formalin-fixed paraffin-embedded blocks from the transurethral resection of bladder tumour of the first diagnosis of HG T1 of the selected patients were retrieval from our institutional pathology archives. All procedures were performed between March 1993 and April 2015. After single pathologist revision of all slides and tissue quality assessment, we retained a final cohort of 86 patients (64 non-progressors and 22 progressors) (S1 Fig). There were no significant differences in clinical and pathological variables between progressors and non-progressors, except for the presence of carcinoma in situ (CIS) (p = 0.02) (Table 1). Slides were stained for FAP and CK5-GATA3 (double stain), using validated protocols on the Ventana Discovery Ultra and Ventana Benchmark XT platform (S1 Table). Stained slides were scanned and all HG T1 tumour regions within each patient were digitally annotated on a serial H&E-stained slide by an expert uro-pathologist (TG). Multiple tumour stages (T1, Ta or CIS) and/or regions of interest (ROIs) were often present within one patient, but only HG T1 ROIs were used for our analyses. Digital annotations of ROIs were transferred to the image analysis software Visiopharm, and stains were scored via a quantitative method per ROI. CK5 and GATA3 were analysed in the tumour area of HG T1 ROIs, and were measured as relative marker area (RMA) (formula: IHC-positive tumour area/total tumour area). FAP was analysed in the stromal area of HG T1 ROIs, and was also measured as RMA (formula: IHC-positive stromal area/total stromal area; Fig 1). Ethical approval was obtained at the Ethics Committee Research UZ/KU Leuven (internal study number S59191) in accordance with the ethical standards of the Declaration of Helsinki of 1964 and its later amendments, with a waiver of informed consent due to the retrospective nature and the secondary use of tissue.

**Table 1:** Clinical and pathological characteristics of included patients.

	All patients		Non-progressors		Progressors		*P-value* *
	n = 86		n = 64		n = 22		
	*No*.	*%*	*No*.	*%*	*No*.	*%*	
Gender							
Male	69	80	49	77	20	91	0.1
Female	17	20	15	23	2	9	
Age							
<60 years	13	15	12	19	1	5	0.2
60–70 years	29	34	19	30	10	45	
>70 years	44	51	33	52	11	50	
Smoking history							
Active	12	14	8	13	4	18	0.8
Never	36	42	27	42	9	41	
Stopped	38	44	29	45	9	41	
T1 status^&^							
Primary T1	71	83	54	84	17	77	0.4
Secondary T1	15	17	10	16	5	23	
Prior intravesical therapy							
BCG	1	1	0	0	1	5	0.2
Chemotherapy	4	5	3	5	1	5	
Size of lesions (cm)							
<3	31	36	22	34	9	41	0.6
≥3	55	64	42	66	13	59	
Number of lesions (n)							
≤3	39	45	28	44	11	50	0.6
>3	47	55	36	56	11	50	
Presence of CIS	25	29	14	22	11	50	0.02
T1 substage^#^							
extensive invasion	51	59	39	61	12	55	0.6
Microinvasion	35	41	25	39	10	45	
Presence of detrusor	73	85	52	81	21	95	0.1
Re-TURBT performed							
Yes–before BCG ind.	24	28	16	25	8	36	0.6
Yes–after BCG ind.	53	62	41	64	12	55	
No	9	10	7	11	2	9	
Detrusor at re-TURBT if absent at T1 TURBT (n = 13)							
Present detrusor	4		3		1		0.2
Absent detrusor	6		6		0		
No re-TURBT	3		3		0		
BCG							
Induction (≥5/6)	86	100	64	100	22	100	
Maintenance (≥2/3 1^st^)^%^	23	27	20	31	3	14	0.1

*Abbreviations*: BC: bladder cancer; CIS: carcinoma in situ; ind.: induction; HG: high grade; LG: low grade; NMIBC: non-muscle-invasive bladder cancer; ROIs: regions of interest; TURBT: transurethral resection of bladder tumour.

*Comparison between progressors vs. non-progressors by chi-squared test.

^&^Primary T1: no prior NMIBC; Secondary T1: prior Ta and/or Tis NMIBC;

^#^Lamina propria invasion depth cut-off 0.5mm.

^%^Adequate BCG as defined by the FDA.

## Results

**Fig 2:**
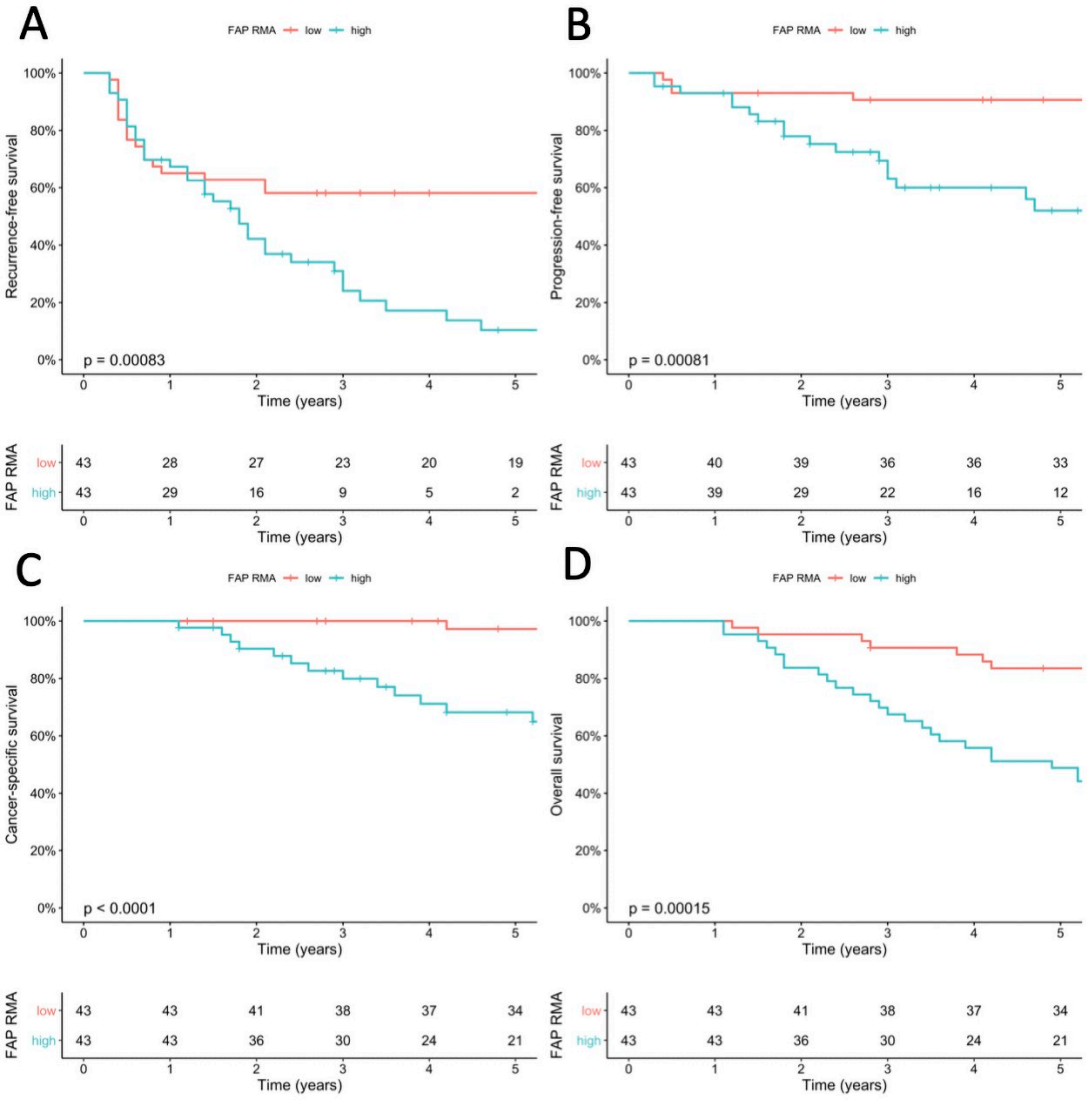
Kaplan-Meier plots of clinical outcome stratified by FAP expression as relative marker area (RMA) in HG T1 regions of interest (low [≤median] vs. high [>median]). FAP expression stratified all patients (n = 86) significantly for each of the investigated clinical outcomes. A: Recurrence-free survival; B: Progression-free survival; C: Cancer-specific survival; D: Overall survival.

**Fig 3:**
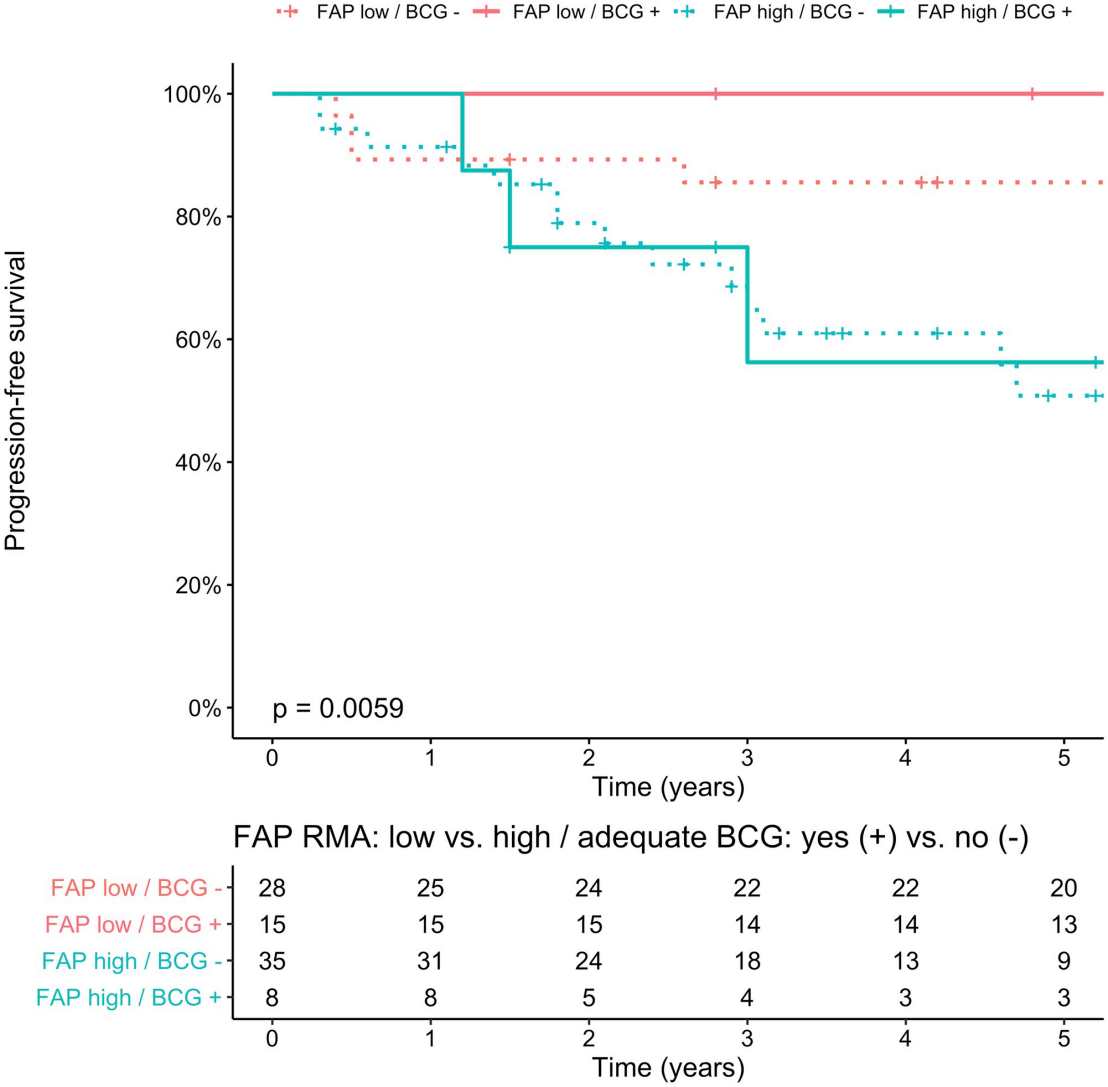
Kaplan-Meier plot of progression-free survival (PFS) stratified by FAP expression as relative marker area (RMA) (low [≤median] vs. high [>median]) in T1 regions of interest, and adequate BCG (as defined by FDA; yes vs. no). Four groups are visualized: FAP low with inadequate BCG (n = 28), FAP low with adequate BCG (n = 15), FAP high with inadequate BCG (n = 34), and FAP high with adequate BCG (n = 8). All patients received al least By performing a pairwise comparison between groups using log-rank corrected by Bonferroni for multiple testing, we identified significant differences in PFS between: 1. FAP low with adequate BCG *vs*. FAP high with inadequate BCG (p = 0.02), and 2. FAP low with adequate BCG *vs*. FAP high with adequate BCG (p = 0.04). However, no significant differences were found between 1. FAP low with adequate BCG *vs*. FAP low with inadequate BCG (p = 0.5), and 2. FAP high with adequate BCG *vs*. FAP high with inadequate BCG (p = 1).

**Fig 4:**
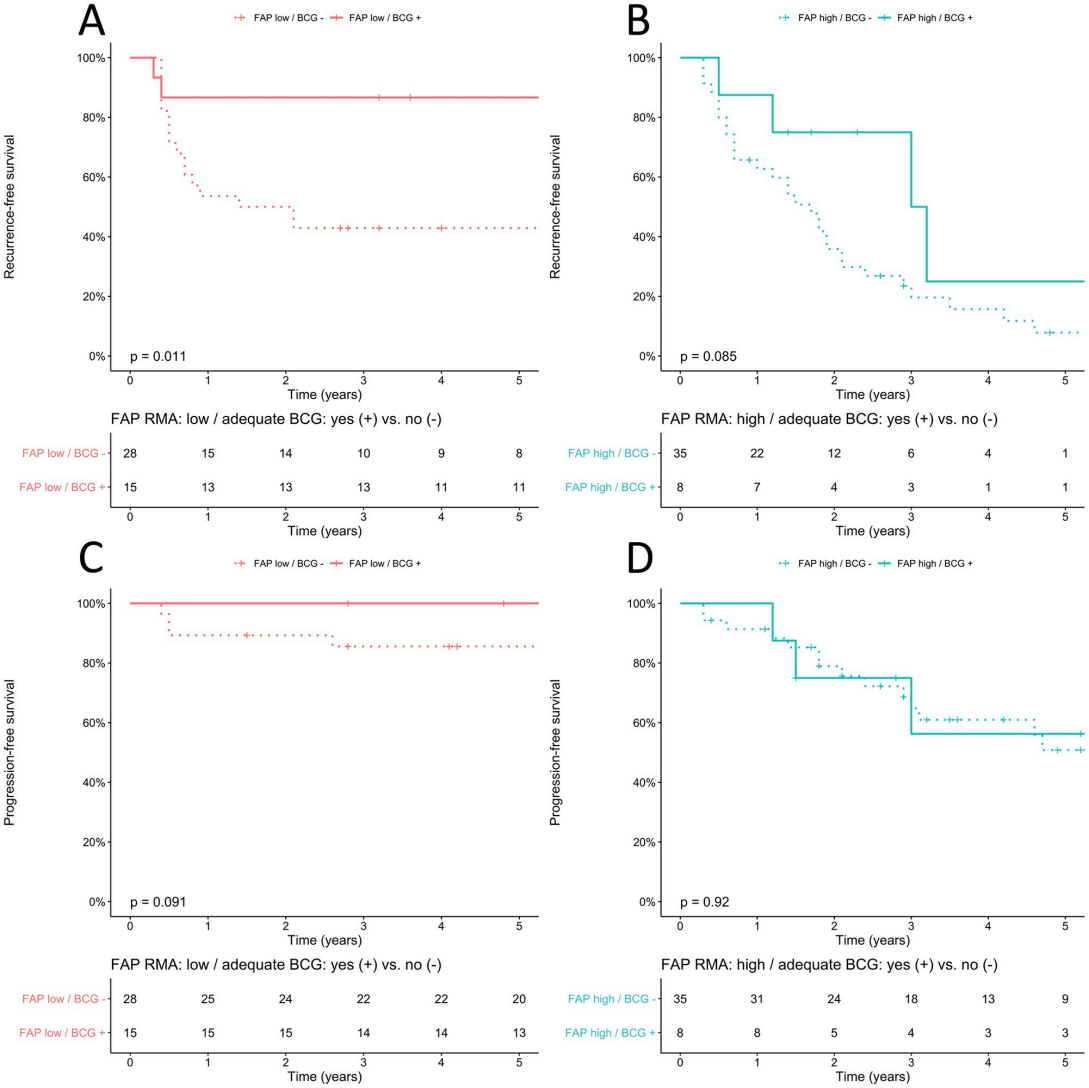
Kaplan-Meier plot of recurrence-free survival (RFS), or progression-free survival (PFS) stratified by adequate BCG (as defined by FDA; yes vs. no). Four groups are visualized: A: RFS for FAP low stratified by adequate BCG (n = 43), B: PFS for FAP high stratified by adequate BCG (n = 43), C: PFS for FAP low stratified by adequate BCG (n = 43), D: PFS for FAP high stratified by adequate BCG (n = 43). There was a significant difference in RFS in FAP low patients stratified by adequate BCG (yes vs. no; p = 0.01).

The 5-year progression-free survival (PFS) of the total population was 73% (95% Confidence Interval [CI]: 64–84). Median time to progression was not reached, and median follow-up of non-progressors was 7.8 years (Interquartile range [IQR]: 4.7–11.7). FAP RMA was significantly higher in HG T1 ROIs of progressors *vs*. non-progressors (p<0.05). There were no significant differences between progressors and non-progressors in CK5 and GATA3 RMAs in T1 ROIs. There were no significant differences in FAP, GATA3 and CK5 RMAs between patients with a primary diagnosis of HG T1 with no prior NMIBC (primary T1; n = 71), and a secondary diagnosis of T1 with prior Ta and/or Tis NMIBC (secondary T1; n = 15). We constructed a bivariable Cox proportional hazards model with FAP RMA in HG T1 ROIs, and adequate BCG (per Food and Drug Administration [FDA] definition: at least 5/6 doses of induction plus 2/3 doses of maintenance, or at least 5/6 doses of induction plus at least 2/6 doses of a second induction course; yes vs. no) for recurrence-free survival (RFS), PFS, cancer-specific survival (CSS), and overall survival (OS). CUETO progression score was not included in the model as our primary cohort was pair-matched using this score, and CUETO progression scores remained balanced between groups following single pathologist revision of all slides and tissue quality assessment (p = 0.8) [9]. There was no significant difference in RFS (p = 0.08) or PFS (p = 0.8) when stratifying for presence of CIS (yes vs. no). Additionally, there was no correlation between FAP RMA in HG T1 ROIs and CUETO progression score (ρ = -0.02; p = 0.8), and CUETO progression score was also not prognostic for PFS (p = 0.5), and RFS (p = 0.2). Interestingly, FAP RMA in HG T1 ROIs was prognostic for RFS, PFS, CSS, and OS in these bivariable models (for PFS: S2 Fig). CK5 and GATA3 were not prognostic for these endpoints in comparable bivariable models replacing FAP with either CK5 or GATA3. Adequate BCG, as defined by the FDA, only impacted RFS, but not PFS, CSS, and OS, which stresses the fact that FAP is indeed an independent prognostic variable for all endpoints. To visualize the impact of FAP RMA in HG T1 ROIs, we dichotomized FAP as a categorical variable and compared low vs. high using log-rank test with the median FAP RMA as cut-off (Fig 2). Additionally, we visualized PFS stratified by FAP RMA in T1 ROIs (low vs. high) and adequate BCG (yes vs. no), which suggested a potential predictive role of FAP (Fig 3). However, no significant differences in pairwise comparison were found in PFS in *FAP low* patients between adequate vs. inadequate BCG (p = 0.5), and in *FAP high* patients between adequate vs. inadequate BCG (p = 1). We did find a significant difference in RFS in *FAP low* patients between adequate vs. inadequate BCG (p = 0.01) on separate analyses for RFS and PFS for *FAP high* and *FAP low* patients (Fig 4). These findings suggest a potential predictive role with regard to RFS of FAP expression for adequate BCG treatment per FDA definition.

## Discussion

This is the first time that a stromal factor is shown to be prognostic for progression of T1 NMIBC. Additionally, luminal and basal markers were not prognostic for clinical outcome in T1 NMIBC. The stromal marker FAP is an emerging biomarker and has been linked to impaired prognosis and therapy resistance in several cancer [2–4]. Moreover FAP-specific PET represents a promising imaging modality for radiotherapy planning [10], and various approaches to clinically target FAP are currently being studied in other cancers [4]. At present, the possible role of FAP in BC is largely understudied. The single arm, phase 2 ABACUS-trial with neo-adjuvant atezolizumab in patients with MIBC found that FAP expression remained high in relapsing tumours whereas a decrease was seen in responders, suggesting that FAP is linked to resistance to therapy [11]. Another study on a mixed MIBC/NMIBC population identified a FAP-dominant cluster prognostic for decreased OS [12], and an additional study on MIBC found that presence of FAP in CAFs implies worse CSS [13]. Our data, focussing exclusively on a HG T1 population, are in line with these findings, strongly suggesting a prognostic role for FAP expression. Clinical response to PD-L1 inhibitors in patients with metastatic UC is associated with a CD8+ immune phenotype in the tumour cells, whilst lack of response is associated with presence of CD8+ immune cells in the fibroblast-rich peritumoural stroma, excluded from tumour cells [14]. Future research regarding FAP in T1 disease should focus on this interaction, for example by assessing tumor infiltrating lymphocytes or CD8+ cells in tumour cells and the peritumoural stroma, and their correlation with FAP [15]. Strengths of this study are the pair-matched design, analysis of an exclusive T1 NMBIC cohort, and the detailed dissection and inclusion of all HG T1 ROIs within each single patient. FAP expression in HG T1 ROIs remained strongly prognostic for RFS, PFS, CSS, and OS in a bivariable model corrected for adequate BCG. Limitations are its retrospective design, a relatively small sample sizes, and absence of detrusor at re-TURBT in a limited number of patients (n = 9). FAP might serve as an easily applicable prognostic biomarker to risk-stratify patients with HG T1 NMIBC if these results are prospectively validated in a larger series.

## Supporting information

S1 Fig Selection flowchart of included patients diagnosed with high-grade T1 NMIBC (n = 120). *T1 patients were pair-matched in 1:3 for CUETO progression score variables. ^$^Patients were excluded due to: T1-absent: absence of T1 on the serial slides following the initially evaluated clinical H&E slide; T2: presence of T2 disease at revision; T0: absence of relevant tumour at revision; Low quality: FAP stain was of low quality with no more left-over slides. ^#^After revision of slides, patients were still matched for CUETO progression score variables (p = 0.8). ^%^Primary T1: no prior NMIBC; Secondary T1: prior Ta and/or Tis NMIBC.
(TIF)

S2 Fig Forest plot of bivariable Cox proportional hazards model for progression-free survival (PFS) incorporating FAP expression as relative marker area (RMA) in T1 regions of interest (per factor 10; continuous variable), and adequate BCG (as defined by FDA; yes vs. no). (TIF)

S1 Table Properties of the antibody clones used. (DOCX)

## References

[1] Bessa A, Maclennan S, Enting D, Bryan R, Josephs D, Hughes S, et al. Consensus in Bladder Cancer Research Priorities Between Patients and Healthcare Professionals Using a Four-stage Modified Delphi Method. European Urology. 2019 Aug;76(2):258–9. doi: 10.1016/j.eururo.2019.01.031 30712969

[2] Liu F, Qi L, Liu B, Liu J, Zhang H, Che D, et al. Fibroblast activation protein overexpression and clinical implications in solid tumors: a meta-analysis. PLoS One. 2015;10(3):e0116683. doi: 10.1371/journal.pone.0116683 25775399 PMC4361589

[3] Wynn TA, Ramalingam TR. Mechanisms of fibrosis: therapeutic translation for fibrotic disease. Nat Med. 2012 Jul 6;18(7):1028–40. doi: 10.1038/nm.2807 22772564 PMC3405917

[4] Fitzgerald AA, Weiner LM. The role of fibroblast activation protein in health and malignancy. Cancer Metastasis Rev. 2020 Sep;39(3):783–803. doi: 10.1007/s10555-020-09909-3 32601975 PMC7487063

[5] Mhawech-Fauceglia P, Yan L, Sharifian M, Ren X, Liu S, Kim G, et al. Stromal Expression of Fibroblast Activation Protein Alpha (FAP) Predicts Platinum Resistance and Shorter Recurrence in patients with Epithelial Ovarian Cancer. Cancer Microenviron. 2015 Apr;8(1):23–31. doi: 10.1007/s12307-014-0153-7 25331442 PMC4449344

[6] Zhang M, Xu L, Wang X, Sun B, Ding J. Expression levels of seprase/FAPα and DPPIV/CD26 in epithelial ovarian carcinoma. Oncol Lett. 2015 Jul;10(1):34–42. doi: 10.3892/ol.2015.3151 26170973 PMC4487033

[7] Kamoun A, de Reyniès A, Allory Y, Sjödahl G, Robertson AG, Seiler R, et al. A Consensus Molecular Classification of Muscle-invasive Bladder Cancer. European Urology. 2020 Apr 1;77(4):420–33. doi: 10.1016/j.eururo.2019.09.006 31563503 PMC7690647

[8] Dadhania V, Zhang M, Zhang L, Bondaruk J, Majewski T, Siefker-Radtke A, et al. Meta-Analysis of the Luminal and Basal Subtypes of Bladder Cancer and the Identification of Signature Immunohistochemical Markers for Clinical Use. EBioMedicine. 2016 Oct;12:105–17. doi: 10.1016/j.ebiom.2016.08.036 27612592 PMC5078592

[9] Fernandez-Gomez J, Madero R, Solsona E, Unda M, Martinez-Piñeiro L, Gonzalez M, et al. Predicting nonmuscle invasive bladder cancer recurrence and progression in patients treated with bacillus Calmette-Guerin: the CUETO scoring model. The Journal of urology. 2009 Nov;182(5):2195–203. doi: 10.1016/j.juro.2009.07.016 19758621

[10] Windisch P, Zwahlen DR, Koerber SA, Giesel FL, Debus J, Haberkorn U, et al. Clinical Results of Fibroblast Activation Protein (FAP) Specific PET and Implications for Radiotherapy Planning: Systematic Review. Cancers (Basel). 2020 Sep 15;12(9). doi: 10.3390/cancers12092629 32942573 PMC7564725

[11] Powles T, Kockx M, Rodriguez-Vida A, Duran I, Crabb SJ, Van Der Heijden MS, et al. Clinical efficacy and biomarker analysis of neoadjuvant atezolizumab in operable urothelial carcinoma in the ABACUS trial. Nature Medicine. 2019;25(11):1706–14. doi: 10.1038/s41591-019-0628-7 31686036

[12] Mezheyeuski A, Segersten U, Leiss LW, Malmström P-U, Hatina J, Östman A, et al. Fibroblasts in urothelial bladder cancer define stroma phenotypes that are associated with clinical outcome. Sci Rep. 2020 Jan 14;10(1):281. doi: 10.1038/s41598-019-55013-0 31937798 PMC6959241

[13] Calvete J, Larrinaga G, Errarte P, Martín AM, Dotor A, Esquinas C, et al. The coexpression of fibroblast activation protein (FAP) and basal-type markers (CK 5/6 and CD44) predicts prognosis in high-grade invasive urothelial carcinoma of the bladder. Hum Pathol. 2019 Sep;91:61–8. doi: 10.1016/j.humpath.2019.07.002 31279874

[14] Mariathasan S, Turley SJ, Nickles D, Castiglioni A, Yuen K, Wang Y, et al. TGFβ attenuates tumour response to PD-L1 blockade by contributing to exclusion of T cells. Nature. 2018 Feb 14;554(7693):544. doi: 10.1038/nature25501 29443960 PMC6028240

[15] Hendry S, Salgado R, Gevaert T, Russell PA, John T, Thapa B, et al. Assessing tumor infiltrating lymphocytes in solid tumors: a practical review for pathologists and proposal for a standardized method from the International Immuno-Oncology Biomarkers Working Group. Adv Anat Pathol. 2017 Nov;24(6):311–35. doi: 10.1097/PAP.0000000000000161 28777143 PMC5638696

