# Supplementary figures and images for: Stromal marker fibroblast activation protein drives outcome in T1 non-muscle invasive bladder cancer
Source: PLoS One. 2021 Sep 15;16(9):e0257195. doi: 10.1371/journal.pone.0257195 (PMC8443055; doi:10.1371/journal.pone.0257195)

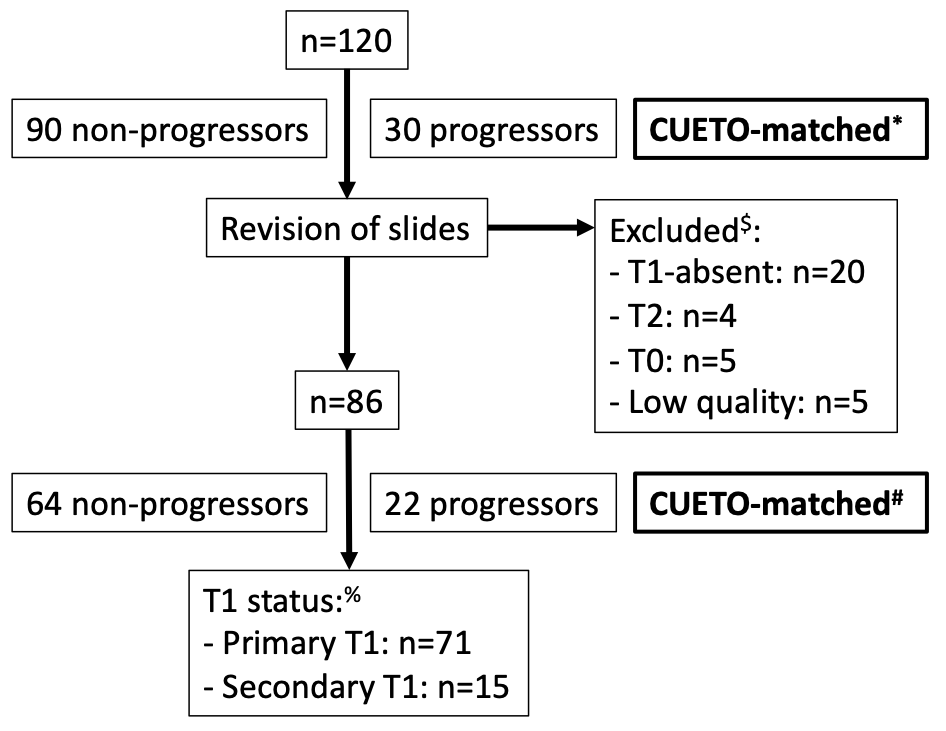

Supplement: S1 Fig — *T1 patients were pair-matched in 1:3 for CUETO progression score variables. $Patients were excluded due to: T1-absent: absence of T1 on the serial slides following the initially evaluated clinical H&E slide; T2: presence of T2 disease at revision; T0: absence of relevant tumour at revision; Low quality: FAP stain was of low quality with no more left-over slides. #After revision of slides, patients were still matched for CUETO progression score variables (p = 0.8). %Primary T1: no prior NMIBC; Secondary T1: prior Ta and/or Tis NMIBC. (TIF) [file pone.0257195.s001.tif]

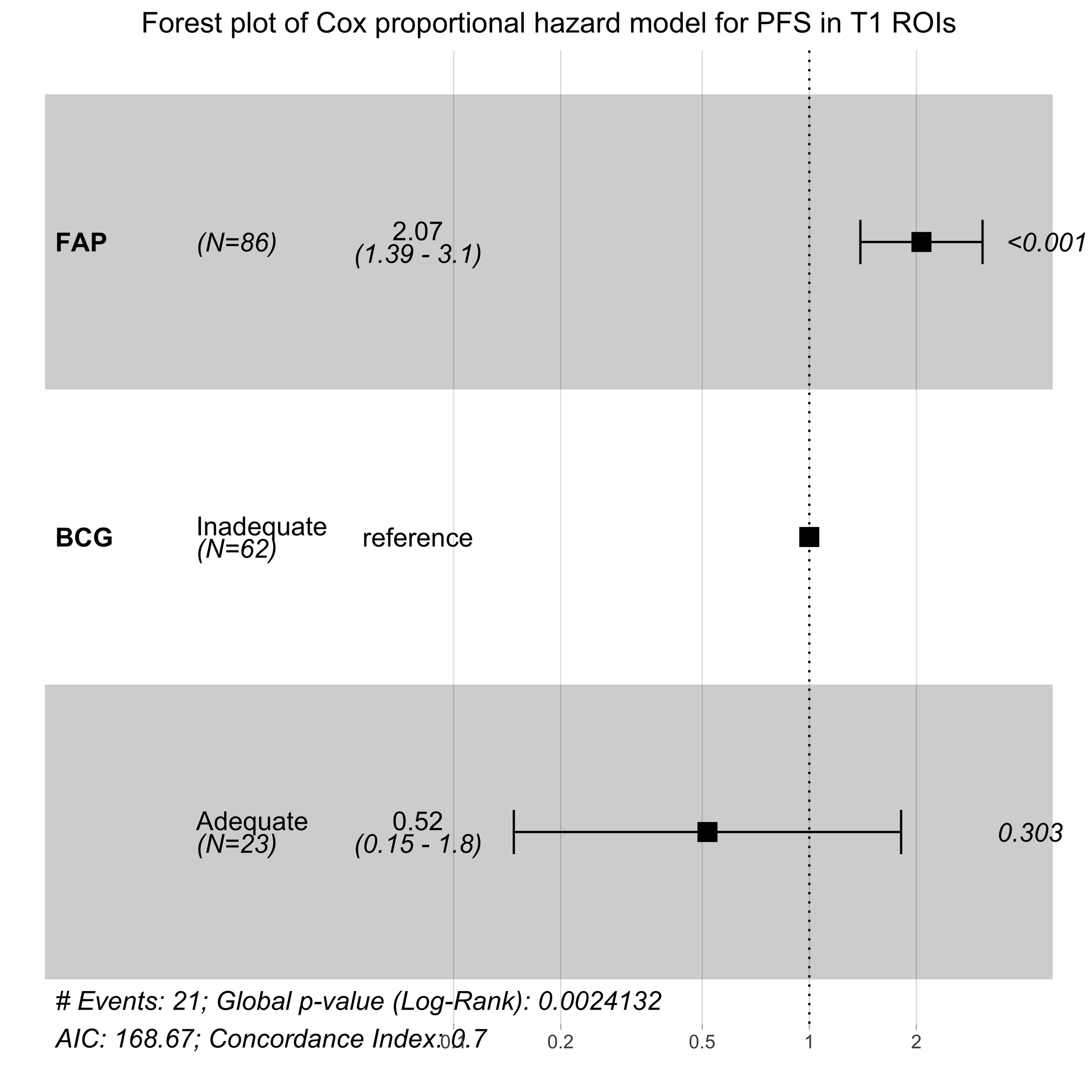

Supplement: S2 Fig — (TIF) [file pone.0257195.s002.tif]
