# Supplementary material for: Stromal marker fibroblast activation protein drives outcome in T1 non-muscle invasive bladder cancer
Source: PLoS One. 2021 Sep 15;16(9):e0257195. doi: 10.1371/journal.pone.0257195 (PMC8443055; doi:10.1371/journal.pone.0257195)
Supplement: S1 Table — (DOCX) [file pone.0257195.s003.docx]

**S2 Table:** Properties of the antibody clones used

| **Immunogen** | **Clone** | **Manufacturer/Code** | **Host** |
| --- | --- | --- | --- |
| *Fibroblast activation protein-α (FAP)* | SP325 | Spring Bioscience M6250 | Rabbit |
| *Cytokeratin 5 (CK5)* | SP27 | Ventana 760-4935 | Rabbit |
| *GATA binding protein 3 (GATA3)* | L50/823 | Ventana 760-4935 | Mouse |
